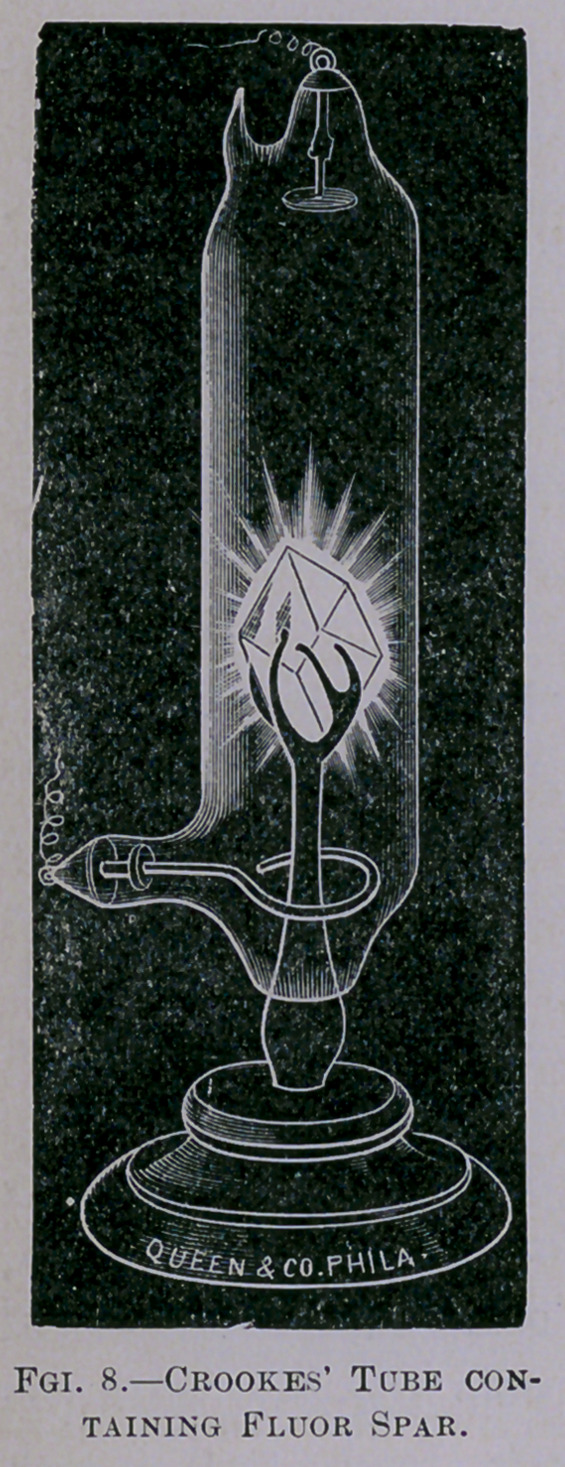# Editorials

**Published:** 1896-03

**Authors:** 


THE
### HOMŒOPATHIC PHYSICIAN,
A MONTHLY JOURNAL OF
HOMŒOPATHIC MATERIA MEDICA AND CLINICAL MEDICINE.

"If our school ever gives up the strict inductive method of Hahnemann, we are lost, and deserve only to be mentioned as a caricature in the history of medicine."—CONSTANTINE HERING.

Vol. XVI. MARCH, 1896. No. 3.

### EDITORIALS.

ARSENICUM-ALBUM.—Having been requested to give the key-notes of Arsenicum as they stood in Dr. Guernsey's *Obstetrics*, the Editor has thought fit to comply with the request, and accordingly, instead of giving notes upon some other remedy, he simply inserts the valuable key-notes, with the admonition to the reader that he commit them to memory. They are as follow:

Sexual desire with an involuntary discharge of mucus as a particular symptom; great restlessness; thirst for cold water, but a very little satisfies; unhappy fatiguing dreams; nothing comes out right in her dreams; erectile tumors with burning lancinating pains; the varicose veins burn like fire; prolapsus vaginæ; the parts have a black look and burn like fire; great anguish with great restlessness; lancinations from the abdomen into the vagina; leucorrhœa whilst standing and emitting flatulence; leucorrhœa thick and yellow, corroding parts touched by it; burning, throbbing, lancinating pains; burning like fire; fear of death; she is sure she will die; cold water aggravates her symptoms; sensitive to cold; wants more covering over her; wants to be wrapped up; hysterical asthma developed at every little excitement. Symptoms always worse at night, especially the latter part of the night; cannot lie down for fear of suffocation; cancer of the uterus with lancinating pains; terrible dartings and lancinatings which burn like fire. Acrid, corroding, burning uterine discharges thick or thin, of a brown or black color and very offensive. Patient is easily fatigued (gangrene of the uterus); rapid sinking; cold perspiration. Undigested food passes through the bowels with great distress and increase of prostration. Water disagrees with the stomach; it does not pass out from it, but seems to lie there and distress her; legs seem almost paralyzed; she can hardly walk; she is very weak and wearied by exertion. Intense burning or tensive pains in ovaries; some relief from constantly moving the feet; pains relieved by motion; ovarian dropsy, the swelling being either small or enormous and the patient completely anasarcous. Sleep full of tiresome dreams; menorrhagia in feeble females; cachectic, affected with rheumatism, and disorganization of uterus and ovaries. Eruptive fevers when aphthæ break out; metrorrhagia; very little appetite; disgust for fat food, butter, meat, dishes of meal and flour, and sweet things. Desire for acids, bread, brandy, coffee, milk. Pale, white face. Bitterness in the mouth after eating and drinking; sensation of a stone in the stomach; nightly vomiting; vomiting of fluids as soon as taken; exhausting diarrhœa, containing undigested food. Very weak; least motion fatigues her very much; very offensive diarrhœa renewed after eating and drinking; burning and shooting pains; heat and agitation on going to stool; painful constriction immediately above the anus which extends toward the sacrum. After stool anus burns like fire, causing intense agony, restlessness, and exhaustion. Heat and pain in the rectum with a kind of tenesmus, as in dysentery, with continual pressure.

The child cries much during and after nursing, or as soon as it takes food; emaciation of babies; aphthæ of children assuming a blue or livid appearance. Offensive and painful stool after taking nourishment; stool and vomiting at the same time; coldness of extremities. Scarlet fever; the eruption disappears too quickly, and the throat becomes putrid; general anasarca. Measles with much prostration and great heat of the skin. Urticaria when apparently caused by unsuitable food. Crusta lactea very dry and scaly, even seems to cause destruction of the hair in the affected places: dry scaly dandruff, scurf, or scales constantly falling off. Symptoms all aggravated by the least exercise; cold sweat; dry scald head; yellow skin; convulsions; the child lies as if dead; it is pale but warm; it is breathless for some time, finally it twists its mouth from one side to the other; a violent jerk appears to pass through the body, and its respiration and consciousness gradually return. These spasms return at longer or shorter intervals until death occurs.

---

THE RŒNTGEN RAYS.*—So much has been published in the daily papers upon the subject of the wonderful Rœntgen Rays that any attempt upon the part of the Editor of this journal to add anything to the explanations already given must seem in the highest degree superfluous and as wasting valuable space that should be reserved for subjects more nearly of interest to the profession.

Yet the tone of the communications received at this office indicates that there is still some misapprehension, some want of understanding of the origin of these rays, which would indicate that there have been some omissions on the part of the published articles upon this subject.

Hence, the Editor is inspired to give his own version of the subject, in the hope that he will be able to clear up these hazy points in the minds of those who do him the honor to read THE HOMŒOPATHIC PHYSICIAN.

To get a clear mental picture of what has been discovered we must first consider the spectrum.

When a beam of sunlight is admitted through a hole in a shutter into a room that is dark, and is allowed to fall upon a three-cornered piece of glass commonly called a triangular prism, the beam passes through the prism, and on emerging upon the other side it is decomposed and separated into seven colors. This is the celebrated experiment and discovery of Sir

---

*The illustrations used in this article are furnished by courtesy of J. W. Queen & Co., Philadelphia.

Isaac Newton, and must be familiar to every reader of these lines.

By placing a white screen before the prism the colors will fall upon it, and may then be studied at leisure.

By allowing the colors as they issue from the prism to fall upon a large lens, they can be again combined together into white light.

If the axis of the prism be placed vertically, and the beam of sunlight permitted to fall upon it, we will have a long, narrow image on the screen, like a ribbon, in which the red will be toward the left, and the colors will fall in the following order: Red, orange, yellow, green, blue, indigo, and violet.

If a thermometer, of exquisitely delicate construction, and called among physicists a "thermo-electric pile" (Fig. 1), be applied to these colors with a view of determining their temperature relatively to each other, it will be found that there is no heat in the violet, nor in any other colors, except the red. Here the heat is manifested by the delicate instrument mentioned above, and on moving the instrument through the red color, still further to the left into the dark space beyond, there is a higher degree of heat than there is in the red space. This heat space is called the Infra-red, calorific, or thermal space.

FIG. 1.—THERMO-ELECTRIC PILE.

Conversely, if a photographic plate be put upon the screen so that all these seven colors may fall upon it and affect the sensitive chemicals with which it is charged, it will be found that there is no appreciable effect of the red upon the plate; nor of the other colors until we arrive at the blue, when we notice that the plate begins to darken, and the shade gradually increases through the indigo and violet, until, in the dark space beyond the violet, the plate acquires its deepest shade, showing that there is some influence there which the eye does not perceive but which is perceived, so to speak, by the photographic plate.

The dark space, which is pervaded by this chemical influence, is called the actinic or ultra-violet space.

This ultra-violet or actinic space, is perhaps the most wonderful region of the whole spectrum. Here resides an influence which, while perfectly invisible to the eye, is capable of decomposing salts of silver, and throwing down the silver as a black powder, in other words, producing photographic action. It is also capable of producing the phenomenon called "fluorescence." This is a property possessed by certain chemicals of absorbing light from any given source, storing it up, and then emitting it again in the dark with a faint glow. The property is possessed by a variety of substances, among which may be mentioned Chlorophyl, or green coloring matter of leaves and grasses; extract of horse-chestnut bark and sun-flowers; Thalline, from petroleum; Sulphate of Quinine, when dissolved in a solution of Tartaric Acid; Salts of the metal Thallium; Nitrate of Uranium and Uranium-glass, which is glass to which Uranium has been added, giving it a canary-yellow color, with a suspicion of green. It is frequently seen in the household as an ornament on the mantelpiece. These substances fluoresce brilliantly in the ultra-violet space above mentioned. Finally, in this ultra-violet space, resides the extraordinary influence now known as the "Rœntgen Rays," or "X Rays," or "Cathode Rays."

The accepted view of the nature of light is the "undulatory theory." According to this theory the whole universe is pervaded by an exceedingly attenuated mobile gas or fluid called the luminiferous ether, not perceptible except when thrown into vibrations of many millions of waves in a second, and, consequently, affecting the nerves of feeling, wherefore we call it heat. A higher rate of vibration affects the eye, and we call it light. If the light come from a solid body, as from molten iron or from the fine particles of carbon in an ordinary lamp or candle flame, or even from a white-hot poker, the vibrations are of all rates of frequency, and so *all* the colors of the spectrum are given off, but, being intimately mixed, appear as white light, or, at most, with a yellow tinge. A prism, however, will show that all the colors are present.

If the light come from a highly heated or incandescent gas, or from a metal heated to a state of vapor, then the light will

FIG. 2.—TŒPLER-HOLTZ ELECTRICAL MACHINE.

be of only one or two colors and the spectrum will have only a few colored lines in it, with much dark space between. The place which these lines will hold in the colors of the spectrum will depend upon their vibratory capacity, and hence these colored lines afford a means of identifying the metal or gas, and, consequently, we have the science of spectrum analysis.

If these explanations have been properly understood, one branch of the subject has been learned, and we are now prepared to inquire into the manner in which the Rœntgen rays are produced.

For this purpose it will be necessary to consider an ordinary lightning stroke. We all know how, when lightning descends from the sky to the earth, it proceeds in zig-zag lines. The usual explanation for this peculiarity is that when the lightning starts out on its trip to the earth it condenses the air immediately in advance of it, and so creates a resistance, which it avoids by darting to one side. In this direction a new resistance is created, and it again changes its course to avoid the new

FIG. 3.—RHUMKORFF COIL.

obstruction. Thus its course to earth is continually hampered, and from this cause its path is so irregular.

Such being the action of lightning, the inquiry is naturally suggested: How would the lightning behave if sent through rarefied air, and what would be the effect of sending it through a vacuum? Artificial lightning strokes, infinitely less powerful than the natural phenomenon, may be produced in the laboratory. For this purpose there are two general classes of apparatus. One instrument used is a rotating glass disk, a modification of the old friction machines, improved by various experimenters—Carré, Holtz, Tœpler, and others (Fig. 2). This instrument gives sparks several inches in length, resembling a miniature lightning stroke.

Another instrument is the Induction Coil, or Faraday or Rhumkorff Coil (Fig. 3), which is only an enlarged form of the small coil seen in so many physicians' offices for producing the Faradic current. This instrument will give a spark, according to the capacity for which it is built, from a quarter of an inch to a yard in length, and with a sharp sound in the largest machines like the crack of a rifle many times repeated. For the purpose of experiment, such an electrical current may be

FIG. 4.—AIR-PUMP WITH BELL-GLASS RECEIVER ON THE PLATE.

passed into a bell glass located on the plate of an air-pump so that the air may be exhausted (Fig. 4).

Another form for producing the same effect is the electrical egg (Fig. 5), which may be attached to the plate of the pump in place of the bell-glass. When full of air, if the electric spark be passed through the vessel, there is no difference in its action from what is observed outside of it. If the air be partially withdrawn by working the air-pump, the spark is observed to become broader, more nearly a straight line, and to make less noise. If the air be withdrawn to the full capacity of the air-pump, so as to make almost a vacuum, there is no longer any spark to be seen; but the whole vessel is pervaded with a soft purple light of unexampled beauty. If a wine-glass, having a few scraps of tin foil in its bottom, be placed upon the plate of the air-pump (Fig. 4), and covered with the bell-glass receiver, and the electric current passed into the receiver so as to enter the wine-glass, the purple light seems to enter the glass, well up within it, as if it were wine being poured in, and finally to overflow it on all sides in a miniature cascade—Gassiott's Cascade. This phenomenon must be seen to be appreciated. If the wine-glass be colored with Uranium—the canary-yellow glass before spoken of, the phenomenon is more magnificent.

FIG. 5. ELECTRICAL EGG.

Another step was taken in the investigation of these phenomena by Dr. Geissler, of Bonn, Germany, who constructed tubes of various lengths from six inches to a yard in length, and of a diameter from half an inch to two inches. These tubes were hermetically sealed; were exhausted of air, almost but not quite completely, and were fitted with platinum wires at either end, melted into the glass, so that one end of the platinum wire was extended within the tube a quarter of an inch, and the other end was in the outer atmosphere.

Thus it was possible to pass an electrical current from an induction coil through the rarefied atmosphere of these tubes from one end to the other. The result was a brilliant light, which was of a lovely purple color, if the remnant of gas sealed up in the tube were ordinary air—red, if the tube contained hydrogen gas, and various other colors, if there were other gases present. This is the celebrated Geissler tube, one of the most remarkable inventions of the day.

The magnificence of the display of light and color made by these tubes beggars description. The Fourth of July with its most costly display of fire-works isn't "in it" compared with the glory of a select set of these tubes, manipulated by an expert electrician. They have attracted the universal attention of the learned world, and have been the subject of numberless investigations.

There are many varieties of them, but the principle is the same—a tube which is almost a vacuum, and yet contains a trace of some particular gas, or the salt of some metal, and is provided with wires by which the electric current is enabled to enter and leave the tube at the will of the experimenter.

Tubes made in this way have been subjected to the analytical test of the prism after the manner of Sir Isaac Newton, with the most marvelous results. Lines of color are formed invariably the same for the same chemical compounds, and so enabling the philosopher to use them as a means of testing for the presence of the metals and gases in other unknown bodies, and this method is one variety of the science of spectrum analysis before noticed. The tubes prepared thus for this kind of testing are called Plücker's tubes, from their originator. They are much used in astronomical observatories for comparing with the spectra of the stars of heaven, and so determining the presence in these stars of the various chemical elements that prevade the earth. This is the science of " Stellar Chemistry."

Professor Crookes, of London, the famous editor of the scientific journal called *Nature*, discoverer of the metal Thallium, has spent much time in the investigation of the Geissler Tube, and has discovered some remarkable mechanical, fluorescent and thermal or heat effects from the passage of the electrical current through them. If a terminal wire entering these tubes be furnished with a minute concave mirror then, on connecting it with the induction coil, the electrical current proceeding from the coil into the tube can be concentrated into a cone of rays or focus, which can be made to fall upon a piece of platinum, heating it red hot. It can be made to fall upon a tiny wind-mill

FIG. 6.—CROOKES' TUBE WITH ENCLOSED WIND-MILL.

made of mica enclosed within the tube, causing the mill to rotate with great rapidity (Fig. 6). Professor Crookes constructed a tiny railroad of glass within the vacuum tube; placed upon it a tiny axle furnished with little paddles, like a water-wheel (Fig. 7), and then on sending the electrical current through the tube, the little axle was caused to roll from one end of the tube to the other.

FIG. 7.—CROOKES' TUBE WITH RAILROAD.

These tubes have been named from their inventor, "Crookes' Tubes." They are, however, only a variety of the Geissler tube, differing from the latter in having the rarefaction carried to a very high degree, so that they are estimated to contain only the millionth part of the ordinary pressure of the atmosphere, or as ordinarily expressed, a millionth of an atmosphere, their other difference being in the mechanical apparatus contained within them as before stated. It was while experimenting with these tubes that Professor Rœntgen discovered the X rays.

These rays are found at what is called the *cathode* end of the tube.

The explanation of this term is as follows:

A galvanic battery or Holtz machine or Rhumkorff coil has always two poles, the positive pole and the negative. The wires attached to these two poles for conducting the electrical current elsewhere, are called *electrodes*, from two Greek words, signifying electricity and a way or path—therefore a path for electricity. As the electricity comes *from* the positive pole, that pole, together with the wire proceeding from it, is called the *anode*, from two Greek words, *ana*, up, and *hodos*, way, signifying a way or path up or out. As the electrical current *returns to* the negative pole, that pole, together with the wire attached to it, is called the *cathode*, from two Greek words, *kata*, down, and *hodos*, a way or path; therefore, a path down into the source of the current. Hence, as the phenomena discovered by Professor Rœntgen occurred at the negative or cathode pole, they have therefore been called cathode rays.

Either end of these vacuum tubes may become the cathode pole, according to its attachment to the source of electricity. Consequently, these phenomena can be produced at either end of the tube at the will of the experimenter.

Previously to Professor Rœntgen, the phenomena occurring at the cathode pole of these vacuum tubes were known to other experimenters, especially the capacity of the rays to go through opaque objects. Professor Rœntgen was, however, the first to pass these rays through the human flesh and to display the bones of the living man in the photographs of the hand as published in the newspapers.

FGI. 8.—CROOKES' TUBE CONTAINING FLUOR SPAR.

All the phenomena above referred to as produced in experimenting with these tubes are marvelously curious and beautiful. They must be witnessed to be rightly appreciated.

Let us now consider the rational explanation of the foregoing phenomena, the better to understand the nature of the Rœntgen rays.

Looking first to the phenomena of color in the Geissler and Plücker tubes, it may be concisely stated that the gases therein contained, when excited by the electric current, vibrate, each one at a certain definite rate of speed peculiar to itself, representing a special color, just as a violin string when stretched vibrates at a certain rate that represents some particular note to which it is attuned.

In the case of the Crookes tubes, on the other hand, where the rarefaction is much greater, the particles of air in the tubes are hurled bodily forward from one end of the tube to the other, with such violence that they produce heat of the walls of the tube or of any object within the tubes upon which they are allowed to impinge, just as a piece of iron may be hammered on an anvil until it is red-hot. If allowed to fall upon fluor spar (Fig. 8), various qualities of glass, rubies, emeralds, and diamonds, they cause fluorescence of these substances of marvelous beauty and great brilliancy. In the case of the diamond, the light is fully as bright as a candle, so that a newspaper may be read by its glow.

If allowed to fall upon any mechanical device freely movable, such as a wind-mill, they will cause its vanes to revolve with amazing rapidity, as before stated.

All the apparatus for the display of these brilliant results is exquisitely delicate and beautiful, and may be obtained of James W. Queen & Co., of Philadelphia.

An illustration that has been used in these pages before may be introduced still further to elucidate this projectile action.

If we imagine a cigar-box to be filled with marbles and the lid shut down tightly, on shaking the box, no movement of the marbles occurs and there is no sound.

This would represent the condition of one of these tubes before the air is withdrawn. If we remove some of the marbles and then closing the lid, shake the box, the marbles will move more freely and cause considerable noise when the box is shaken.

If nearly all the marbles are removed, and the lid closed, and the box shaken, the marbles will move with much violence from side to side, with a loud noise, and may even force out the ends of the box. This would represent the condition of the vacuum or Crookes tube at its highest state of rarefaction. If *all* the marbles be taken out of the box, *no* effect follows, and similarly if a Geissler or a Crookes tube be deprived of *every* particle of air, the vacuum be absolute, then no phenomena of any kind are obtained.

With such violence are the air particles hurled at the walls of the vacuum tube that it may be either cracked by the impact or melted by the heat.

Nikola Tesla, the great electrician, finds that the air particles may even be forced through the glass. The impact of these particles against the glass sides of the tube produces a peculiar vibratory motion of the luminiferous ether, by which the vibration is continued through wood, metals, books, flesh, and other opaque objects, and having passed through them is still capable of affecting a photographic plate, and of bringing out fluorescence. This is the celebrated Rœntgen ray, by which photographs of the bones of the living body may be taken at will. Its use in surgery in discovering the location of bullets, needles, and foreign bodies imbedded in the muscular system, and of discovering the character of fractures in bones, has been sufficiently set forth in the daily newspapers.

Think of the idea of photographing through living flesh! Think of photographing through a book of one thousand pages! The mind is staggered by the thought. Were it not for the ocular demonstration of the photographic image, the possibility of such a result would be rejected with contempt and ridicule.

To the homœopathic physician administering his attenuated doses of medicine the Rœntgen ray is peculiarly interesting. The diluted and potentized drugs of our materia medica are so opposed to common sense and all that science has taught and revealed that rational men of learning and common sense have unhesitatingly rejected them and refused all credit to the reports of marvelous cures by their agency. Hahnemannians have therefore despaired of ever convincing the learned world of the truth and justice of their claims for the efficacy of these remedies.

Now in the very midst of this opposition to the homœopathic remedies and the principles upon which they are administered, in the face of the refusal to accept the testimony of its advocates, because, it is loudly declared, the whole system is a violation of common sense and refuted by all the revelations of scientific investigation in other channels, in the face of all this argument, there comes along the wonderful Rœntgen Ray, overturning the entire scientific teaching of the day, and in defiance of the whole formidable army of sceptics in the realm of knowledge, deliberately writing its record in no uncertain hand upon a photographic plate that is concealed under a piece of densest wood; staggering belief and yet compelling it by penetrating sheets of opaque metal, and even whole books!

With such a spectacle as this, the suggestion naturally occurs to the mind that if the adherents of the new school of medicine could but write their record in some similarly vivid way that the opposition of scepticism would melt away, and their art be established upon its proper scientific basis.

There is another thought for the homœopathist. It is found that the Rœntgen ray enables the experimenter to determine the difference between a false pearl and the genuine: between a true diamond and its counterfeit: between true and spurious coins. This suggests that its offices may be invoked for determining the reliability of the potentized remedy and to note any changes in its condition by keeping.

Yet another thought suggests itself. Tesla has sought to photograph the living brain by this wonderful influence. For this purpose the head was subjected to the passage of the rays for a considerable period of time. He reports that the ray produces a sense of fullness and heat.

Why should not the homœopathist seek to procure a proving of the effects of the X-ray upon the animal economy of the human being?

An inviting field is thus opened up to the experimenters of our school. May it soon be cultivated.

## Figures and Tables

**Fig. 1. f1:**
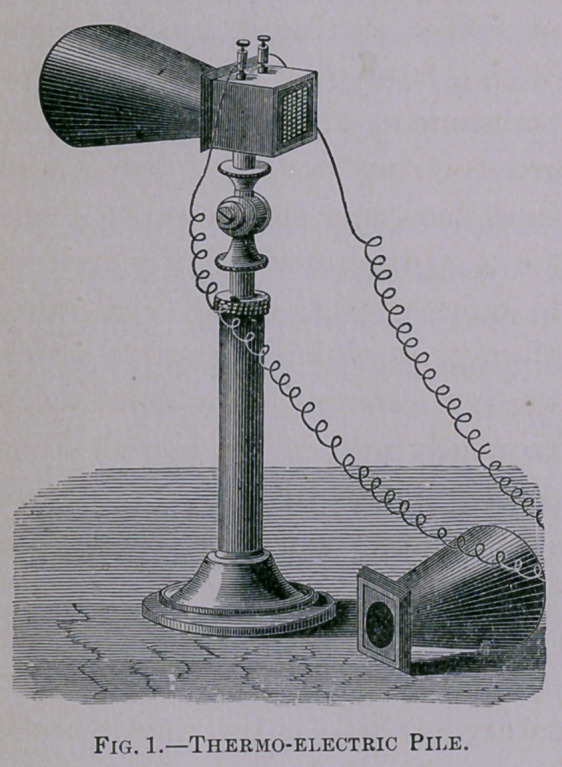


**Fig. 2. f2:**
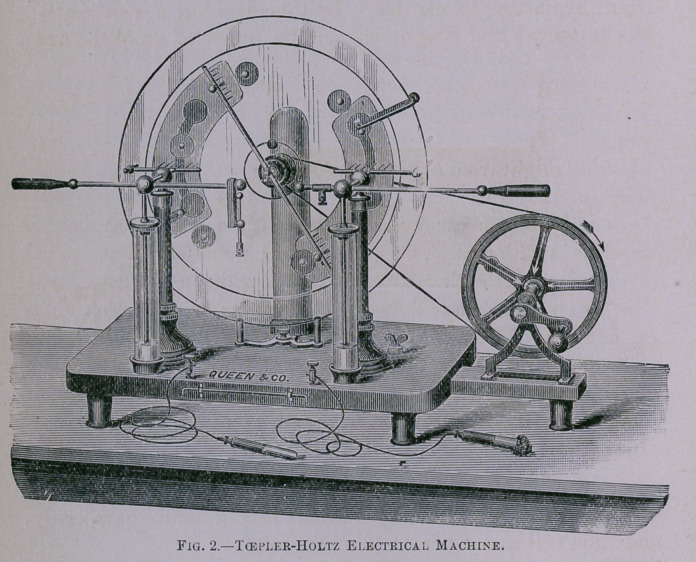


**Fig. 3. f3:**
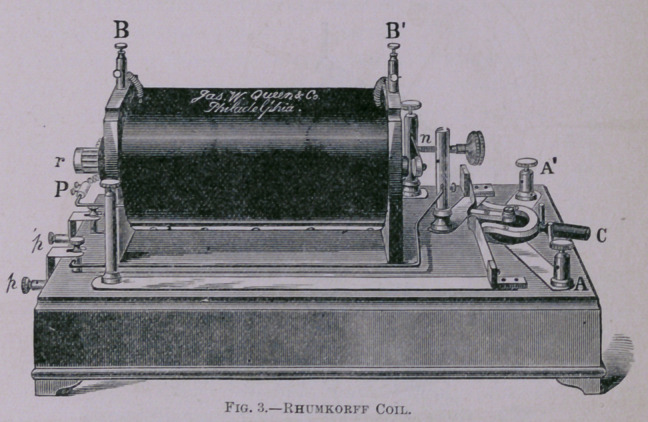


**Fig. 4. f4:**
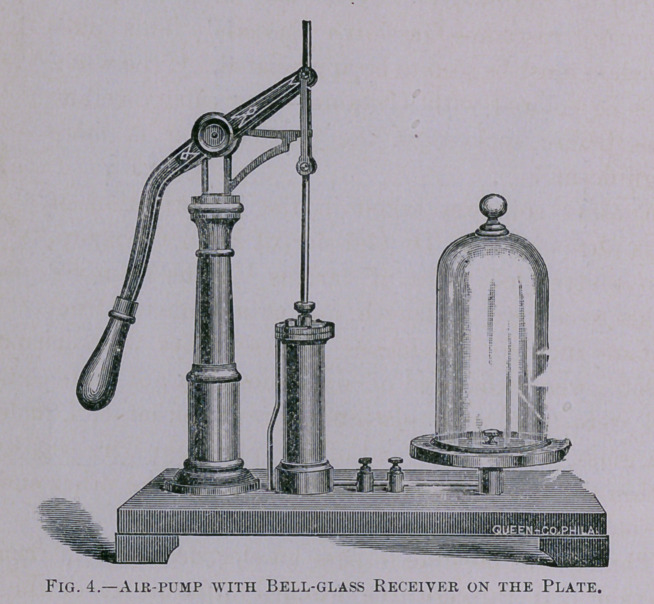


**Fig. 5. f5:**
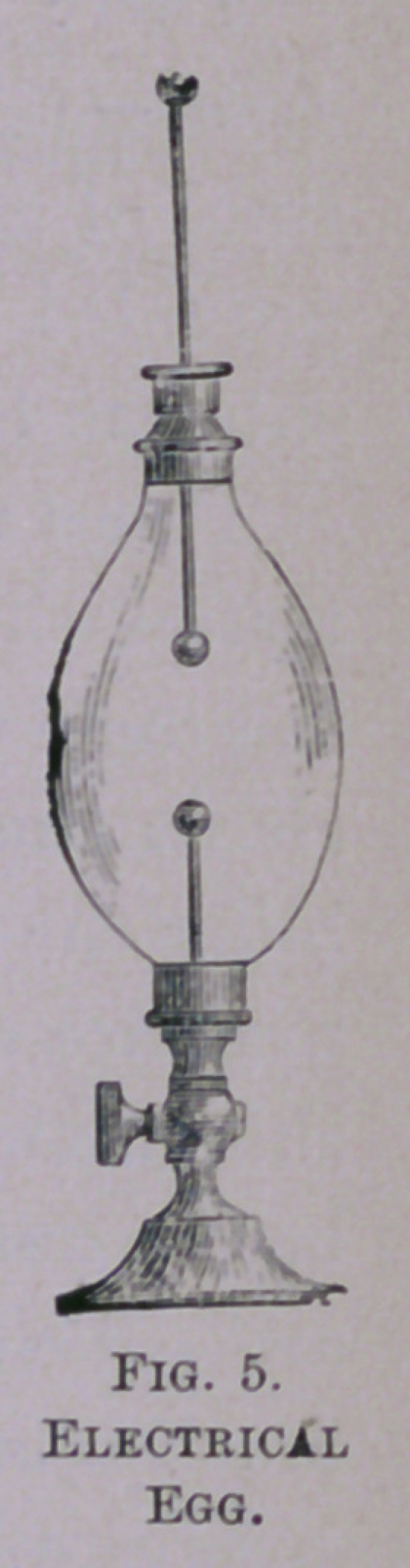


**Fig. 6. f6:**
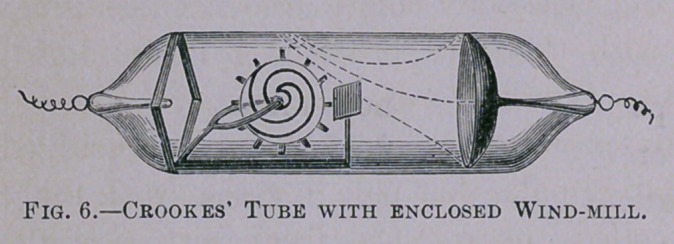


**Fig. 7. f7:**
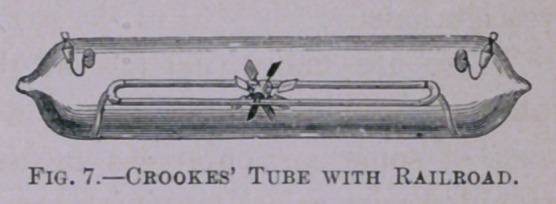


**Fgi. 8. f8:**